# Exploring endophytic bacteria communities of *Vanilla planifolia*

**DOI:** 10.1186/s12866-024-03362-w

**Published:** 2024-06-20

**Authors:** Keshika Mahadeo, Ahmed Taïbi, Jean-Christophe Meile, Bertrand Côme, Anne Gauvin-Bialecki, Hasna Boubakri, Aude Herrera-Belaroussi, Hippolyte Kodja

**Affiliations:** 1 Laboratoire de Chimie et Biotechnologie des Produits Naturels, Faculté des Sciences et Technologies, Université de la Réunion, 15 Avenue René Cassin, CS 92 003, 97 744 St Denis Cedex 9, La Réunion, France; 2 QualiSud, Université de La Réunion, Univ Montpellier, Avignon Université, CIRAD, Institut Agro, Montpellier, France; 3 La Vanilleraie, 2 ter Domaine du Grand Hazier, allée Chassagne, Sainte Suzanne, Réunion, 97441 France; 4 Laboratoire d’Ecologie Microbienne, Université Claude Bernard Lyon 1, UMR CNRS 5557, UMR INRAE 1418, VetAgro Sup, Villeurbanne, 69622 France

**Keywords:** Vanilla accession, Endophytic bacteria, Substrate, Microbial transmission, Curing process

## Abstract

**Background:**

Rhizosphere bacterial community and endophytes are now known to influence plant health and response to environmental stress. Very few studies have reported the diversity of endophytic bacterial communities of *Vanilla planifolia* and their potential roles in promoting plant growth or contributing to aromatic quality.

**Results:**

In this study, the composition and diversity of the Vanilla rhizosphere bacterial community were explored by analyzing rhizosphere soil and root tissue samples as well as green pods of three accessions of *Vanilla planifolia* grown on different types of substrates (compost and leaf litter). In addition, the endophytic bacterial diversity of roots and green pods as well as the evolution of endophytic bacteria after the curing process of vanilla green pods were analyzed based on a metabarcoding approach. The results showed that bacterial species richness and diversity were higher in the compost. The analysis of the soil bacterial composition displayed that *Halomonas, Pseudoalteromonas, Enterobacter* and *Bradyrhizobium* were the most abundant genera. Moreover, the results indicated that the soil bacterial community structure was linked to the host plant genotype. Regarding the roots endophytic bacteria composition, the genera *Halomonas*, *Pseudoalteromonas*, *Bacillus* and *Carboxydocella* genera were present in all samples, independently from the substrate nature. Several genera including *Bacillus*, *Bradyrhizobium, Burkholderia* and *Halomonas* were transmitted internally from the roots to the green pods. The curing process reduced the bacterial richness and bacterial diversity associated with the green pods. *Halomonas*, *Pseudoalteromonas*, *Bacillus*, and *Carboxydocella* are the dominant genera in the pods after the curing process.

**Conclusions:**

This study provides an overview of changes of the bacterial communities dynamics especially endophytic in the roots and the green pods. It highlighted bacterial genera (*Halomonas, Pseudoalteromonas, Bacillus, and Carboxydocella)* potentially implicated in the formation of aroma compounds of vanilla beans.

**Supplementary Information:**

The online version contains supplementary material available at 10.1186/s12866-024-03362-w.

## Background

Vanilla is a tropical orchid, a member of the *Orchidaceae* family [1], . Of the 110 species in the genus, only three species (*V. planifolia, V. tahitensis* and *V. pompona*) are cultivated for their useful flavors and fragrance constituents [1]. However, *V. planifolia* beans dominate the market, accounting for over 95% of the world’s vanilla production [2]. Different cultural techniques are used to cultivate Vanilla, including agroforestry systems, in forest-type land, intensively in deforested land, or in shade houses [3]. On Reunion Island, *V. planifolia* is widely cultivated in forest-type land (95% of the production). The remaining 5% of Reunion Island’s vanilla is grown in shade houses. In forest-type land cultivation, plant nutrition is sourced from terrestrial roots that extract nutrients from decaying leaves around living stakes. Conversely, in the shade house system, plant nutrition is provided by sugarcane compost supplies. Plant quality, including its health and productivity, is strongly influenced by soil composition, especially in the rhizosphere - the zone of soil surrounding the roots. The interactions between plants and the microfauna in the rhizosphere can exert either a positive or negative impact on plant growth [4]. Many rhizobacteria stimulate plant growth (known as Plant Growth-Promoting Rhizobacteria, or PGPR) by modulating plant phytohormone levels or by improving nutrient bioavailability [5, 6].

The soil not only serves as a physical support or a nutrient source for the plant but also hosts a large range of microbial diversity that directly interacts with the root system. These rhizospheric microbial communities, composed mainly of bacterial and fungal populations, take advantage of root exudates, such as sugars, amino acids, and organic acids which serve as a rich source of energy and nutrients. In return, these beneficial microorganisms stimulate plant growth and improve soil health through a variety of mechanisms and various ecological services, including nitrogen fixation, phosphate solubilization, heavy metal sequestration, phytohormones production (i.e. indole acetic acid, gibberellins, or cytokinins), the mineralization of organic matter, nutrient cycling [7–10], as well as creating stress-alleviating enzymes and inducing systemic resistance [11]. In addition, specific root-associated plant growth-promoting microorganisms reduce plant pathogens through several mechanisms, including antibiotics and antifungal metabolites biosynthesis, systemic resistance induction, and siderophore production [12].

Another significant factor impacting vanilla production and the aromatic composition of the beans is the symbiotic relationship between microorganisms and their distribution across various plant tissues, including roots, leaves, and pods. Recently, endophytic bacteria and fungi, including mycorrhizal fungi, have received increased attention due to their potential role in generating specific flavors and fragrances in aromatic plants [1].

Their involvement in the biotransformation of aromatic compound precursors during the cocoa bean fermentation has been evidenced [13]. Similarly, the fungal fermentation of coffee beans highlighted strain-specific variation in aroma content, underscoring their contribution to the formation of aromatic compounds [14]. The importance of the soil rhizosphere microflora in the aroma quality of basmati rice has also been demonstrated [15]. A limited number of studies have focused on the fungal and bacterial communities associated with vanilla beans and their role in aroma profiles [1, 16–18]. It is worth noting that the curing process of vanilla beans involves several treatments such as scalding, sweating, drying, and conditioning. These processes, accompanied by significant temperature variations, are likely to induce changes in microbial communities at each stage. The dynamic evolution of the microbial community during the curing process could potentially influence the aroma profile of the bean [19]. In this context, the current study focuses on three *V. planifolia* accessions, initially selected from forest-type land. These accessions were selected and cultivated in experimental shade houses for their great vegetative growth, flowering potential, and glucovanillin accumulation in mature green pods [20]. Over the past decade, these accessions have been cultivated in experimental shade houses using two types of growing media: litter composed of dead leaves in reference to forest-type land cultivation and compost supplied with sugarcane bagasse regarding shade house cultivation. The three accessions (named as CDY, CHM and CLB) produced high-quality vanilla beans on both growing media (i.e., vanilla beans with high and complex aromatic quality).

This study aims to analyze and compare the composition of bacterial communities present in the rhizospheric soil (compost and litter) with the endophytic flora found in various tissues (roots and beans) of three high-performance vanilla accessions. The main question addressed in the present study was to identify the dynamic of endophytic bacterial communities in different plant organs. To achieve this objective, three specific aspects were investigated:

- the selection of rhizosphere microbiota by vanilla accessions cultivated on two growing media.
- the description of microbiota-transmission from the surrounding soil to the plant roots and thus then to the green beans.
- the influence of the curing process of vanilla beans on the associated bacterial communities.

## Results

### Diversity of soil bacterial communities according to the type of substrate

**Fig. 1:**
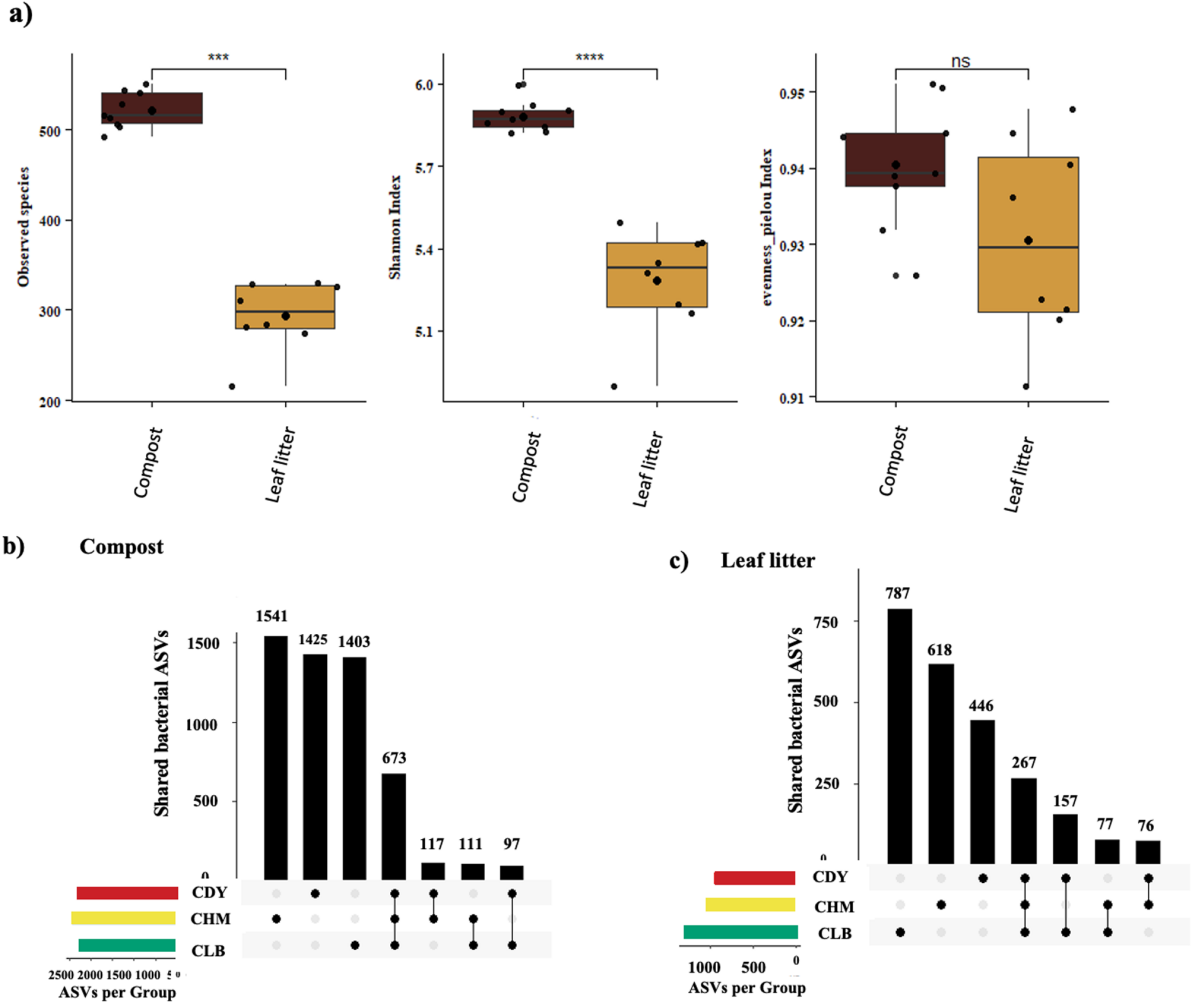
**a**) Boxplot of alpha-diversity in vanilla substrate samples (Compost and Dead leaves “litter” independently from the accessions); from left to right, observed species ; Shannon diversity and Pielou’s evenness indexes were statistically compared between samples. Asterisks indicate significant differences (*p* < 0.05) computed with Wilcoxon rank-sum pairwise test with the false discovery rate (FDR) adjustment. b and c) UpSetR plot of unique and shared bacterial ASVs among the three vanilla accessions (named as CDY, CHM and CLB) samples grown on compost (**b**) and leaf litter substrate (**c**)

**Fig. 2:**
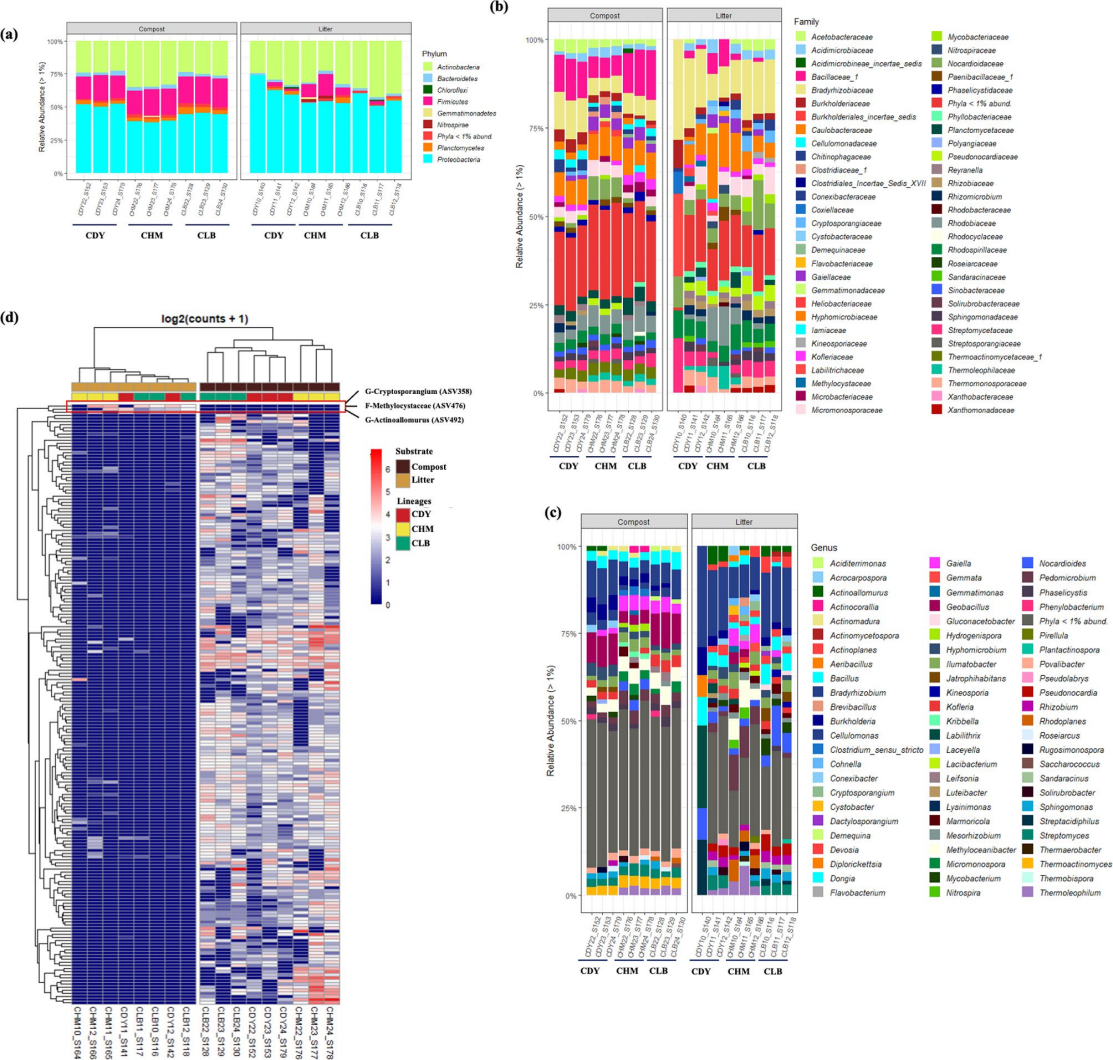
Relative abundance (%) of bacterial compositions at Phylum (**a**), Family (**b**), and Genus (**c**) levels detected in compost and litter substrate samples of vanilla accessions (CDY, CHM and CLB). (**d**) Heatmap of comparison of differential abundance analysis (DESeq2) of bacterial ASVs between vanilla accessions samples grown in compost and litter substrate. Vanilla accessions and growing substrate information are shown with colored bars at the top of the heatmap. Only ASVs with significant differences (P-adjusted < 0.01) in log2 fold change are depicted

Three *V. planifolia* accessions were selected based on their high performance and then grown using two distinct growing media: compost and leaf litter (derived from plant dead leaves). The bacterial communities in both growing media were studied to assess the changes in the microbiota and their potential impact on the quality of vanilla beans. The bacterial community in the rhizospheric soil was inventoried using a metabarcoding targeting V3-V4 16 S rRNA fragment. The distribution of substrate-associated bacteria is depicted in Figs. 1 and 2. A total of 71 genera belonging to 8 phyla were identified in the microbiota of the soil substrate. Alpha diversity metrics revealed that the species richness and diversity are higher in the compost than in the leaf litter (Fig. 1a). In the compost, a total of 5,367 Amplicon Sequence Variants (ASVs) were detected, while 2,428 ASVs were identified in the leaf litter (Fig. 1b and c). Regarding the taxonomic composition, 8 bacterial phyla (> 1% of relative abundance) were detected in both growing media (Fig. 2a). The bacterial composition varied according to the type of substrate: Proteobacteria, Firmicutes, and Actinobacteria collectively made up more than 90% of the total bacterial community in the compost, while the leaf litter contained mainly *Proteobacteria* and *Actinobacteria* as the most abundant phyla; *Firmicutes* was only detected in few samples. At the family level, the bacterial community comprised a total of 57 families, with *Hyphomicrobiaceae* and *Bradyrhizobiaceae* being the most abundant families in both substrates, followed by a high abundance of *Bacillaceae* in the compost (Fig. 2b). The taxonomic composition of the leaf litter at the family level differed essentially by the occurrence of *Phyllobacteriaceae* and *Pseudonocardiaceae*, and the absence of *Cellulomonadaceae*, *Phaselicystidaceae*, and *Thermoactinomycetaceae*. Among the 71 genera detected in the soil microbiota, *Bradyrhizobium* was the most abundant genera in both growing media (Fig. 2c). Moreover, the compost composition displayed higher levels of *Bacillus*, *Gaiella, Geobacillus, Methyloceanibacter*, and *Thermoactinomyces*. When comparing bacterial composition between the two soil substrates, the analysis depicted in Fig. 2d confirmed that the compost exhibited a higher ASVs richness compared to the leaf litter. Besides, both substrates differed from each other by the absence of three specific ASVs in the compost (ASVs 358, 492, and 476) as depicted in the heatmap in Fig. 2d, which belong to the genera *Cryptosporangium*, *Actinoallomurus*, and the family *Methylocystaceae*, respectively.

### Diversity of soil bacterial communities according to vanilla accessions

**Fig. 3:**
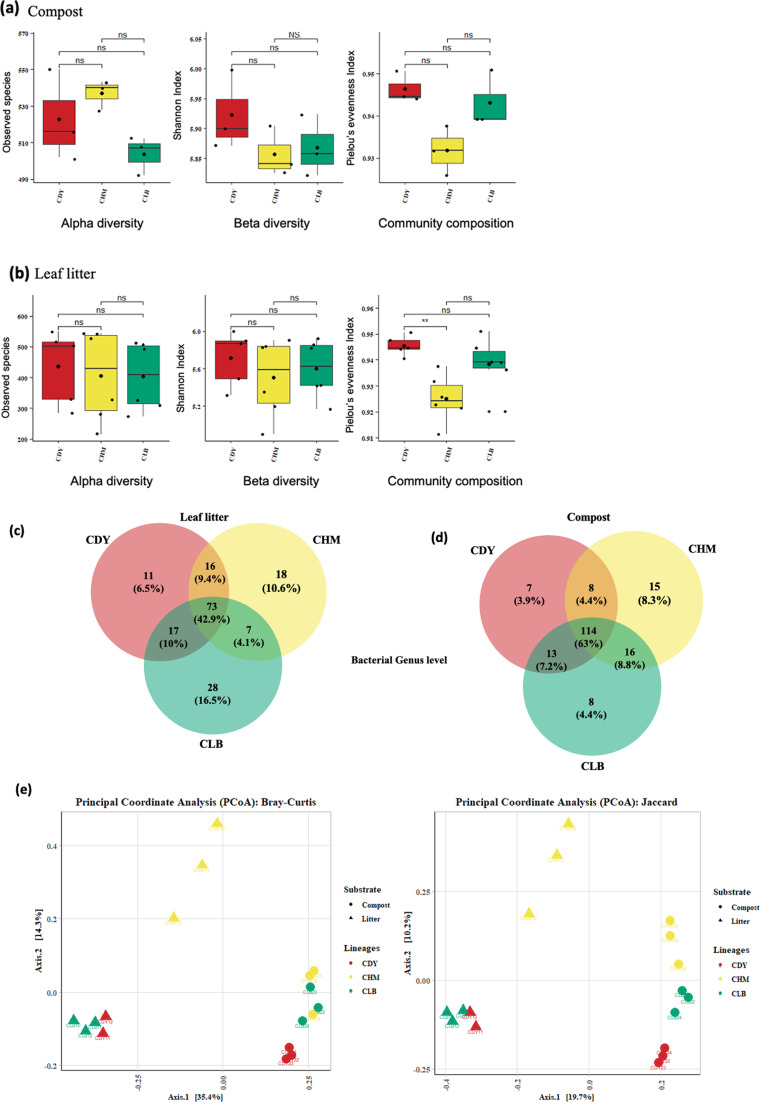
Comparison of alpha, beta diversity, and community composition between soil samples of vanilla accessions cultivated on compost and leaf litter. Boxplots of different alpha diversity indix: Observed species represent community richness; Shannon index represent diversity;, and Pielou’s evenness index represent evenness) based on bacterial communities detected in vanilla accessions cultivated in compost (**a**) and litter (**b**). Asterisks and NS indicate significant differences (p-value < 0.05) and not significant, respectively in Wilcoxon rank-sum pairwise test. Venn diagrams showing the number of unique and shared bacterial ASVs assigned to the Genus level between samples of different vanilla accessions cultivated on Leaf litter (**c**) and Compost (**d**). (**e**) Principal coordinate analysis (PCoA) ordination based on Bray-Curtis and Jaccard (presence and absence of taxa) dissimilarity matrix depicting bacterial structure between vanilla accessions samples: CDY (red), CHM (yellow), and CLB (green) cultivated on Litter (triangle point up) and Compost (point). The x- and y-axes represent the first and second components of the PCoA plot, respectively

The differences in soil bacterial communities within the rhizospheric soil were assessed. The alpha diversity metrics indicated no significant variations in terms of species richness and bacterial diversity across the accession samples cultivated on the same medium (Fig. 3a and b). Therefore, the difference between accessions seemed to have minimal impact on the bacterial communities detected in the soil.

The differences in bacterial community composition between samples belonging to the three vanilla accessions grown in two types of substrates were depicted using Principal Coordinate Analysis (PCoA). The Jaccard index was used to compare bacterial community similarity based on the presence or absence of ASVs, while the Bray-Curtis dissimilarity matrix was employed to compare bacterial community similarity based on ASV abundance. Beta diversity analysis revealed that samples clearly clustered based on the nature of the substrate (Fig. 3e), with the first and second PCoA axes explaining 35.4% and 14.3% of the variance, respectively. This finding was statistically significant (Bray-Curtis, perMANOVA: Pseudo-F = 5.013, *p*-value = 0.001), corroborating the disparities observed in the taxonomic composition of both growing media. In addition, vanilla accessions grown on the compost were grouped, indicating that the composition of soil bacterial communities exhibited similarity regardless of the three accessions. This was confirmed by Pairwise perMANOVA statistical test based on both Bray-Curtis dissimilarity (Pseudo-F = 1.598, *p*-value = 0.026) and Jaccard distance (Pseudo-F = 1.383, *p*-value = 0.022). In contrast, when grown on leaf litter, the accession CHM displayed a significant difference in the relative abundance of the soil bacterial community in comparison to accessions CLB and CDY accessions. As shown in Fig. 3e, CHM samples grown on leaf litter constituted separate a group, separated from other samples. Furthermore, the PCoA based on the Jaccard index indicated that the accession CHM has more specific ASVs present in both growing media. Venn diagrams (Fig. 3c and d) confirmed these findings. They revealed that 114 genera (accounting for 63% of the total detected bacterial community) were common to all accessions grown on the compost, while 73 genera (42.9% of the bacterial community) were shared by the three accessions grown on leaf litter. Vanilla accessions exhibited variations in their soil microbiota selectivity based on the type of growth substrate. Indeed, the three studied Vanilla accessions displayed a higher prevalence of specific genera within their microbiota (6.5%, 10.6%, and 16.5% of total genera for CDY, CHM, and CLB respectively) when cultivated on the leaf litter, in comparison to when grown on the compost (3.9%, 8.3% and 4.4% of total genera for CDY, CHM, and CLB respectively).

**Fig. 4:**
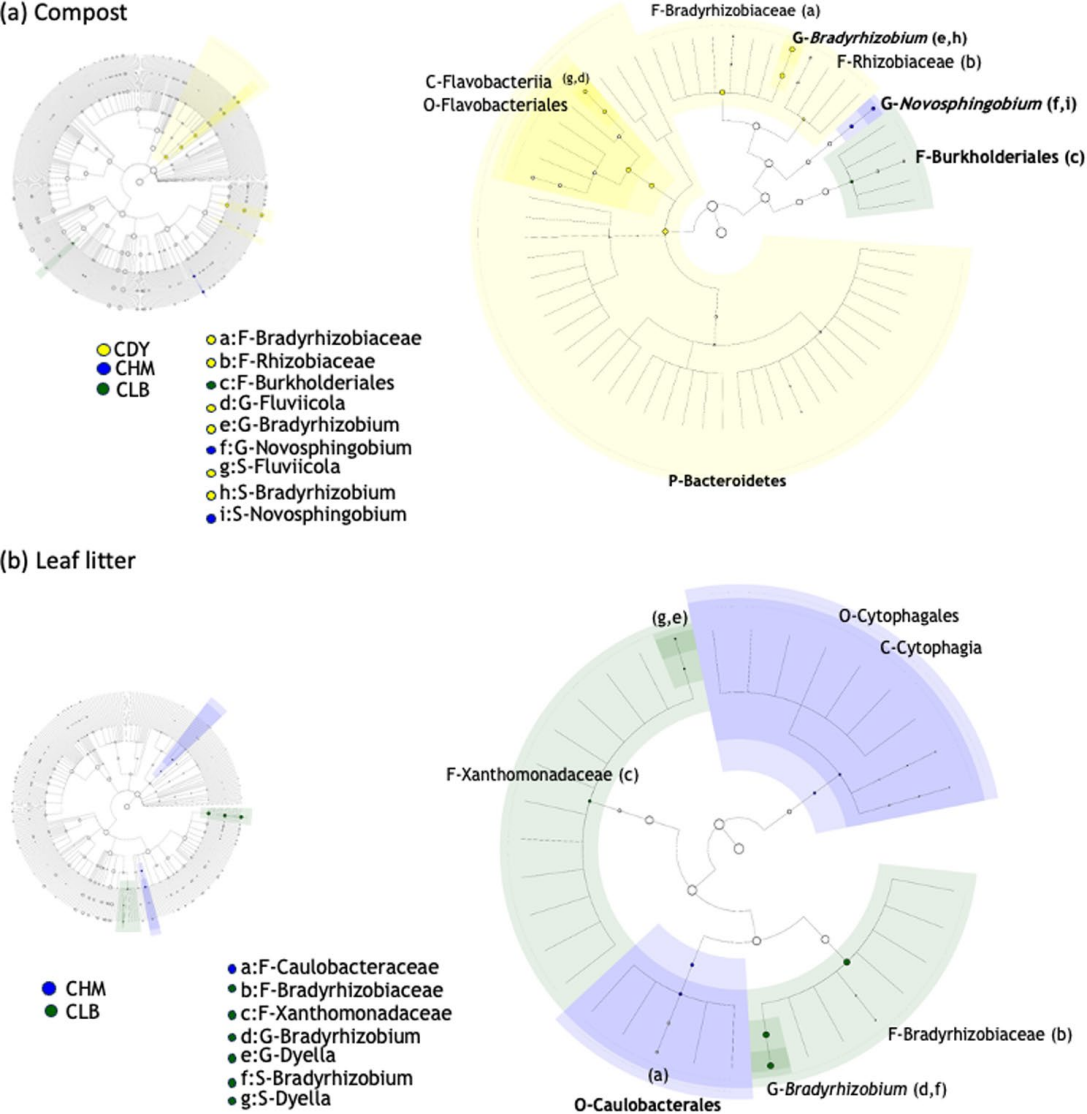
Cladogram generated using the LEfSe analysis based on non-parametric factorial Kruskal-Wallis (KW) sum-rank test (*p*-value < 0.05 ), indicating the phylogenetic distribution of bacterial community associated with the vanilla accessions CDY (yellow), CHM (blue) and CLB (green) grown on compost (a) and leaf litter (b). Only taxa meeting an LDA significant threshold value of > 4 are shown

Considering that bacterial communities associated with vanilla accessions and growth substrates were significantly different, the Linear discriminant analysis Effect Size (LEfSe) was performed to identify differentially abundant species in the vanilla accession samples according to the type of substrates (Fig. 4). The LEfSe algorithm detected 59 differentially abundant taxonomic clades as active biomarkers, which resulted in divergences among the vanilla accessions (CDY, CHM, and CLB) cultivated in different substrates (compost and litter). Specifically, 4 phyla (i.e., *Actinobacteria*, *Bacteroidetes*, *Firmicutes*, and *Proteobacteria*), 11 orders, and 15 families were detected across all samples (Additional Table 1). Independently from vanilla accessions, the comparison of the samples according to the growing conditions showed that the highest number of taxonomic biomarkers was detected in compost considering all vanilla accession samples (37), while the lowest was recorded in litter (22).

In the litter substrate, the genera *Pseudomonas* and *Novosphingobium* were the most abundant in CDY, while *Rhizobium*, *Devosia*, and *Sphingomonas* represented the abundant genera in the accession CHM. However, *Bradyrhizobium* and *Dyella* were the most dominant genera in CLB. As for the compost substrate, in addition to *Thermoactinomyces*, *Fluviicola* were the significantly abundant in the accession CDY, *Microbispora* in CHM, *Flavobacterium* and *Microbispora* in CLB.

### Bacterial transmission from the substrate to roots and green pods

**Fig. 5:**
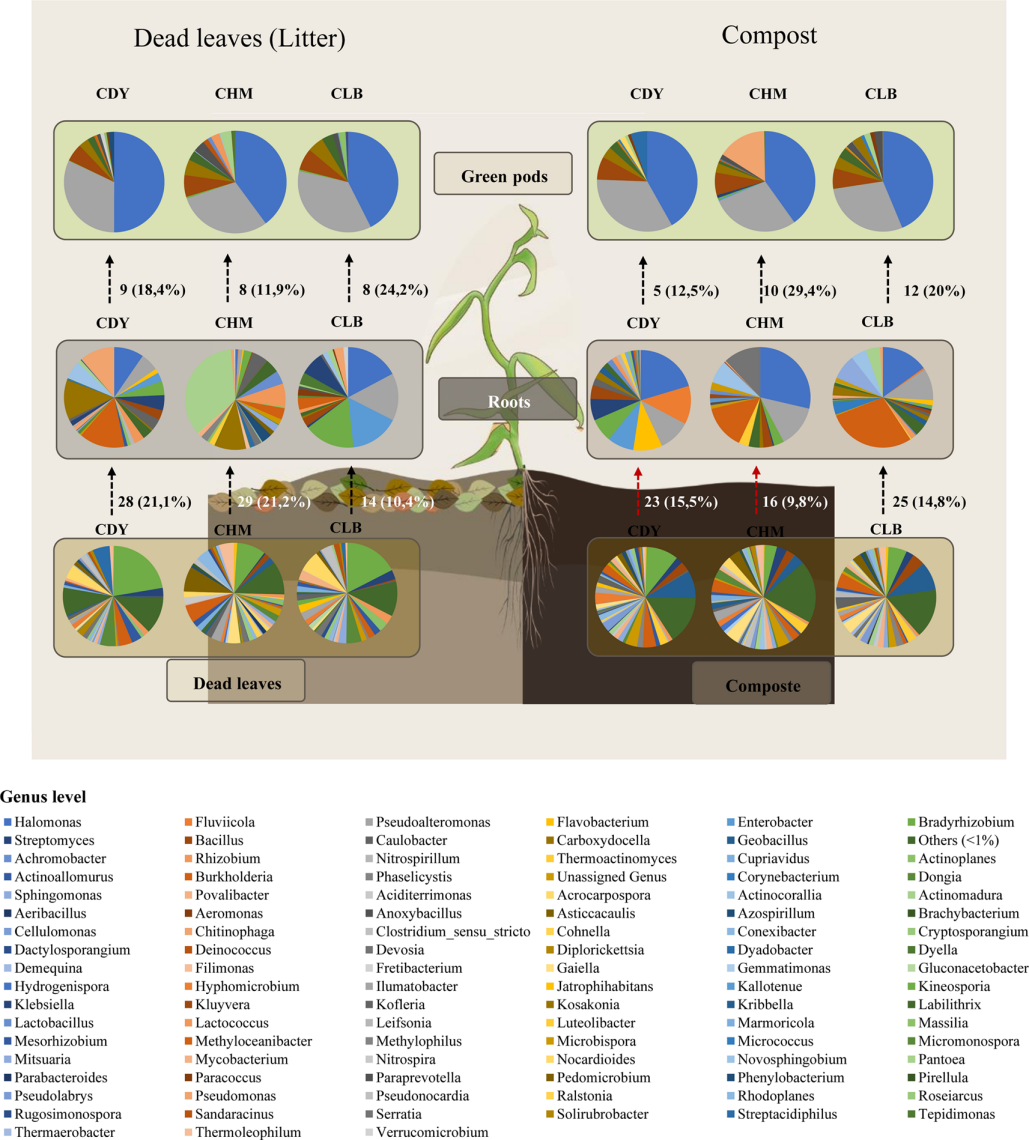
Bacterial diversity (genus level) associated with the three vanilla accessions (named as CDY, CHM, and CLB) grown in two different substrates (Compost and Litter). The bacterial composition of soil as well as endophytic bacteria of plant organs (roots, and green pods) are shown. The figure illustrates bacterial diversity within the substrate used for vanilla cultivation, as well as the transmission of this bacterial community from the substrate to the roots and subsequently to the green pods, categorized based on different vanilla accessions

Endophytic bacterial communities were studied in two organs (roots and green pods) and the internal transmission of endophytic bacteria from the roots to the green pods was investigated. A range of 14 to 29 genera present in the soil, constituting 9.8% (compost) – 21.2% (leaf litter) of the total bacterial flora, successfully colonized the roots of Vanilla plants. Analysis of endophytic bacteria within vanilla roots grown on the compost highlighted the presence of two predominant genera, *Halomonas* and *Pseudoalteromonas*, which were common across all accessions on both substrates (Fig. 5). Nevertheless, the roots from each accession appeared to have a specific endophytic bacteria composition, suggesting that the plant selects its bacterial community. *Burkholderia* is one of the major genera found in root samples for the accessions CLB and CHM grown on the compost. In addition to *Burkholderia*, *Serratia* was one of the most abundant genera in the root samples of CHM while the endophytic bacterial flora of CDY was mainly composed of genera *Fluviicola*, *Flavobacterium*, *Enterobacter*, and *Bradyrhizobium*. A similar observation was made for the plants grown on leaf litter. The endophytic bacterial community of root samples of CLB (when cultivated on leaf litter) was dominated by *Halomonas*, *Pseudoalteromonas*, *Enterobacter*, and *Bradyrhizhobium*. The CHM accession was dominated by genera *Pantoea*, and *Kosakonia* whereas CDY comprised genera *Burkholderia, Kosakonia, Pseudomonas, Halomonas, and Novosphingobium.*

From roots to green pods, the microbiota diversity is reduced. Only 5 to 12 genera, representing 11.9–29.4% of the total microbiota, were common between both organs. Independently from the substrate nature, four genera were abundant in all accessions: *Halomonas*, *Pseudoalteromonas*, *Bacillus* and *Carboxydocella*. The CHM accession differed from the others by the presence and the high abundance of the genus *Pseudomonas* especially when grown on the compost.

**Fig. 6:**
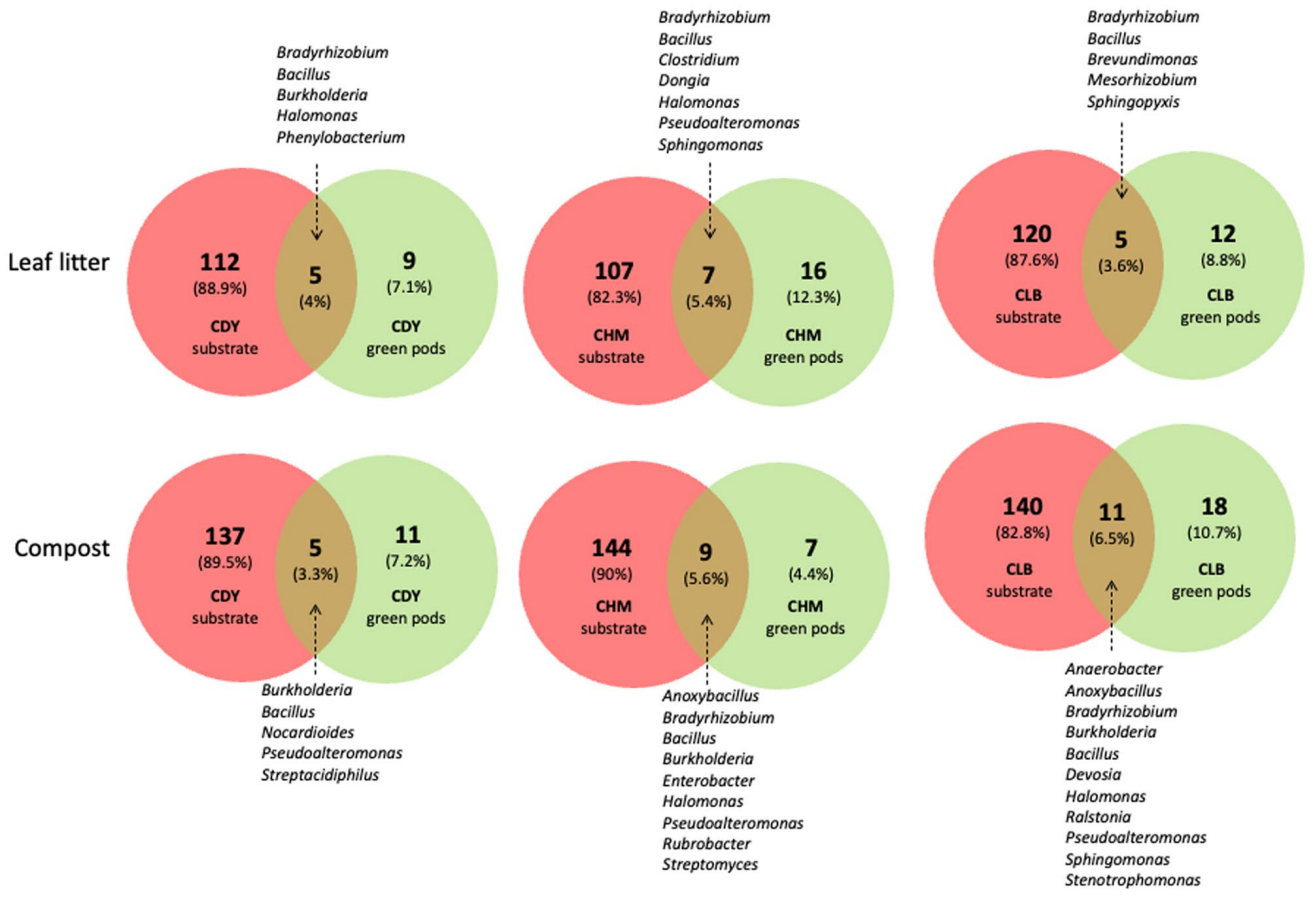
Venn diagrams showing the number of unique and shared bacterial ASVs assigned to the Genus level between the substrate and the green pods of different vanilla accessions (CDY, CHM, and CLB) cultivated on Leaf litter and on Compost

Regarding the bacterial transmission from the substrate to the green pods, a range of 5 to 11 genera, constituting 3.3 to 6.5% of the total bacterial flora identified in the soil, were similarly detected in the green pods (Fig. 6). *Bacillus* is the only genera detected in the substrate to be present systematically in all green pod samples. In addition to *Bacillus*, the genus *Bradyrhizobium* was common to Vanilla accessions grown on the leaf litter while *Burkholderia* was common to those grown on the compost. Besides, the genus *Halomonas* occurred frequently in the green pod samples.

### Diversity of bacterial communities after curing of vanilla

**Fig. 7:**
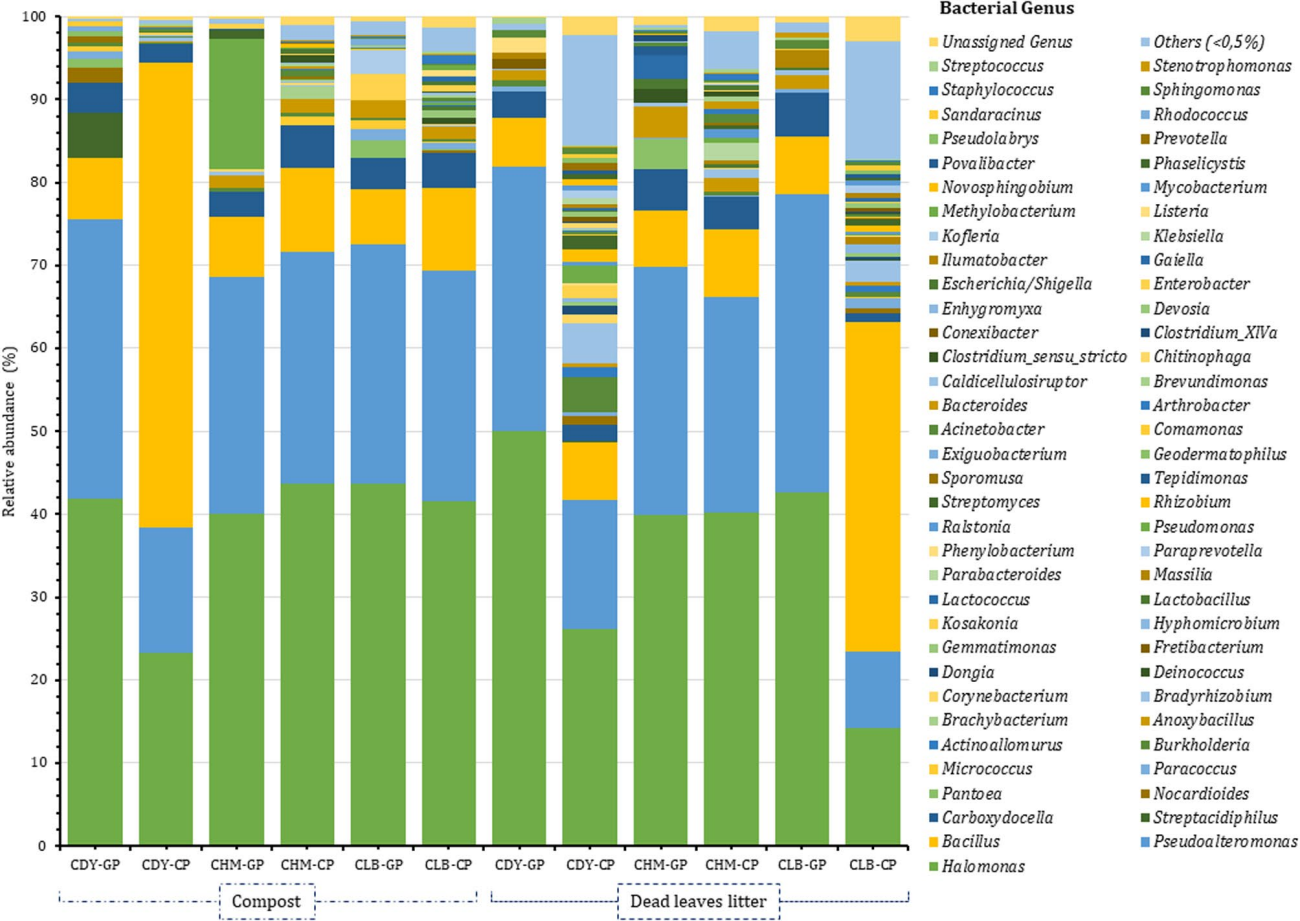
Bacterial composition and relative abundance of bacterial genera in vanilla pods before and after the curing process for accessions CLB, CDY, and CHM. “GP” and “CP” denote green pods and pods after scalding and sweating, respectively

**Fig. 8:**
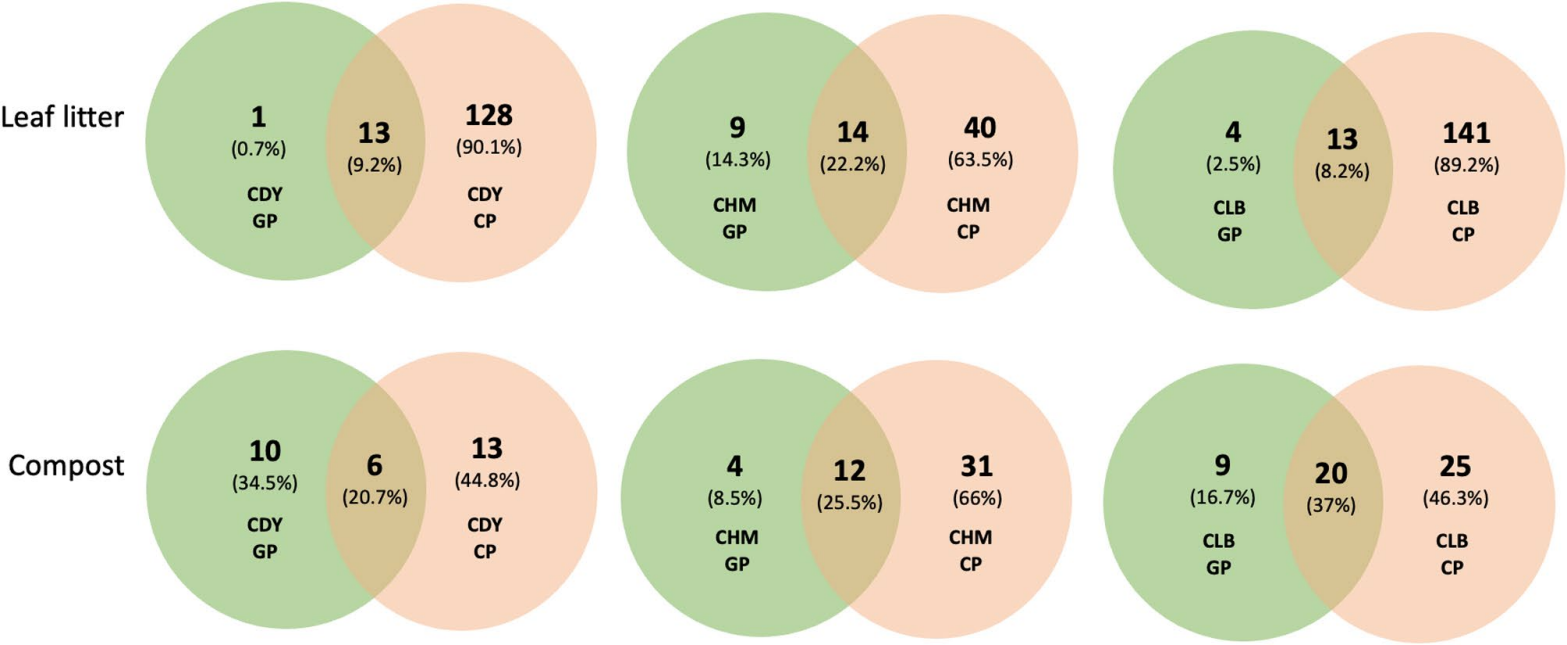
Venn diagrams showing the number of unique and shared bacterial ASVs assigned to the Genus level between the green pods (GP) and pods after scalding and sweating (CP) of different vanilla accessions (CDY, CHM, and CLB) cultivated on Leaf litter and on Compost

The diversity of endophytic bacteria in the pods after scalding and sweating is higher; a minimum of 14 genera were detected in the green pods and a maximum of 158 genera in the pods after the curing process (Figs. 7 and 8). When comparing all accessions cultivated on both substrates, 13 to 141 genera are specific to the pods after the curing process. Besides, a higher diversity of endophytic bacteria was observed in the pods after the curing process when cultivated on the leaf litter. The scalding and sweating steps aim to destroy cell structures and activate the enzyme activity to release glycosides. These factors may be more likely to promote microbial growth, especially thermophilic bacteria, explaining the higher diversity observed after sweating. *Halomonas*, *Pseudoalteromonas*, *Bacillus*, and *Carboxydocella* remain the dominant genera after the curing process representing 51.6% (on beans collected from the accession CDY cultivated on leaf litter) to 99.4% (on beans collected from the accession CDY grown on compost) of the total microbiota. These four genera were present in all accessions on both substrate media and might play a crucial role during the curing of vanilla. CDY (cultivated on compost) and CLB (cultivated on leaf litter) vanilla accessions displayed a different endophytic bacterial community largely dominated by *Bacillus*. In addition, genera *Anoxybacillus* and *Bradyrhizobium* were detected in all samples except the accession CDY obtained from the compost.

## Discussion

The bacterial diversity associated with Vanilla depending on the type of growth substrate was investigated. The results showed that the species richness and diversity were higher in the compost substrate. This difference in total ASVs detected could potentially be due to the greater availability of carbon and nitrogen content in the compost, owing to its supplementation with sugarcane bagasse. Both elements are necessary for biological life development, including bacteria. Regarding the rhizosphere soil composition, the genus *Bradyrhizobium* was predominant in both growing media. Species of this genus are agroecologically important due to their role as symbiotic nitrogen-fixing bacteria. They improve plant nutrient uptake [21] and participate in quorum-sensing mechanisms by producing signaling molecules [22]. Besides, higher levels of *Bacillus*, *Gaiella, Geobacillus, Methyloceanibacter*, and *Thermoactinomyces* were detected in the compost, which is not surprising considering the activity of mesophilic and thermophilic bacteria in the compost. Previous studies have highlighted the use of *Bacillus* for controlling pathogen proliferation [23–25]. *Bacillus* are known to promote plant growth by using many direct or indirect mechanisms such as solubilization and mineralization of nutrients (phosphorus and potassium), nitrogen fixation, the production of phytohormones or siderophores, or by stimulating the host plant defense [26, 27]. Some species belonging to *Gaiella*, *Geobacillus*, and *Thermoactinomyces* genera have been shown to display an important role in biostimulating plant growth and the biocontrol of plant pathogens [28–30].

The soil characteristics and composition can play an important role in shaping the development of selected plant-associated bacteria [31]. Previous studies have indicated that soil microbial diversity is influenced by several factors, including the soil type, the vegetation, the cultural practices, and the plant treatments [32].

The diversity of soil bacterial communities according to Vanilla accessions was investigated and the results showed that the accessions exhibited variations in their soil microbiota selectivity depending on the type of growth substrate. These accessions of *Vanilla planifolia* were selected for their agronomical performance and grew close to each other on different substrates. Soil bacterial communities appear to be dependent on host plant genotype. Different studies have demonstrated that the bacterial profile in the soil depended not only on the plant type [32] but also on the genotype of the plant [33, 34].

The endophytic bacteria composition in the roots was analyzed but we cannot exclude external contaminants. A small portion of DNA from the bacterial communities present on the surface of the tissues may remain even after several washes. Each accession showed a different bacterial profile in the roots according to the type of substrate. It has been reported that root exudates including organic acids, amino acids, and proteins may be involved in recruiting bacterial endophytes from the rhizosphere [35]. The difference in endophytic bacterial community observed for each accession suggested that they have their own composition of root exudates. A quite similar result was obtained for *Arabidopsis* plants grown in different soils which highlighted that the soil type influences the composition of endophytic bacteria found in the host roots [36]. Moreover, it has been demonstrated in *Boechera stricta* that the environmental conditions (soil nutrition, humidity, temperature), host genotype, and age of the plant have a direct influence on root bacterial communities [37].

Regarding the endophytic bacteria composition of the roots, the results showed that 9.8–21.2% of the total bacterial flora present in the soil colonized internally to the roots. The description of the major endophytic bacteria detected (*Halomonas*, *Pseudoalteromonas, Burkholderia, Bradyrhizobium…*) in the roots is consistent with their potential role as Plant Growth-Promoting Rhizobacteria (PGPRs). Most broadly, PGPR includes *Pseudomonas, Aeromonas, Klebsiella, Enterobacter, Azopirillum, Clostridium, Azotobacter, Arthobacter, Rhizobium, Kosakonia, and Bacillus* [38, 39]. Besides, strains from *Halomonas*, *Burkholderia*, and *Bradyrhizobium* have been reported to improve nutrient uptake, N_2_ fixation, promote phytohormones production, and increase plant growth [38, 40]. Furthermore, the genera *Pantoea* and *Serratia* have been reported to induce the systemic resistance of the plant against phytopathogenic bacteria [38, 40]. Thus, the genera that colonized the roots contribute to the nutrient uptake, growth promotion, and resistance of the plant.

Compared to the roots, the microbiota diversity is reduced in the green pods and 11.9–29.4% of the total microbiota were common between both organs. Four genera are predominant in all accessions (*Halomonas*, *Pseudoalteromonas*, *Bacillus*, and *Carboxydocella*) and were transmitted from the roots or the substrate. These endophytic bacterial genera would have acquired the ability to invade the host. They could colonize plant tissues from the roots or through different main entry points including wounds, hydathodes, and stomata [35]. Indeed, the epiphytic and endophytic bacterial communities in *Boechera stricta* were reported to be similar in roots and leaves supporting the hypothesis that the communities are recruited from the soil [37]. Additionally, some endophytic bacteria can modify the plant cell wall by secreting enzymes that facilitate bacterial entry and spread within the plant tissues [41, 42]. Endophytic bacteria live asymptomatically in plant tissues and can play a crucial role in enhancing water absorption to facilitate mineral nutrition or inhibit bacterial and fungal phytopathogens through the production of specialized metabolites, siderophores, or hydrolytic enzymes [43, 44]. Indeed, *Halomonas*, *Pseudoalteromonas*, *Pseudomonas*, and *Bacillus* have been reported to secrete several metabolites that trigger plant growth and prevent pathogen infection [38, 45]. Their presence in green pods would have a preventive effect against phytopathogen attacks. However, a few studies have been reported on the genus *Carboxydocella* and its role here in the green pods remains unclear. Further investigations are necessary to study the involvement of this genus in preventing plant phytopathogen development.

Vanilla pods produce aromas after curing. Vanillin is the main aromatic compound found in vanilla pods and is mostly formed by the hydrolysis of glucovanillin by the enzyme β-D-glucosidase during the curing of green pods. However, Frenkel et al. (2011) have demonstrated that vanillin continued to accumulate in the pods even though the activity of the enzyme decreased during the curing process. In recent years, the role of microorganisms in the formation of aromatic volatile compounds has an increasing interest. Here, we studied the bacterial diversity before and after the key point of the curing process; the scalding of pods in hot water (65–70 °C for 2 min) and the sweating in woolen blankets placed in wooden boxes. The results displayed a greater diversity of specific endophytic bacteria in the pods after scalding and sweating. These findings can be explained by the higher temperature during the sweating offering better conditions for the development of spore-forming and thermophilic bacteria such as *Bacillus* and *Carboxydocella* or other genera that were present in the green pods in low abundance. Furthermore, the four genera (*Halomonas*, *Pseudoaltermononas Bacillus* and *Carboxydocella*) predominant in the green and cured pods could arise from the transmission from the roots or the substrate as they were detected in these samples.

*Bacillus* was found to be the predominant bacterial genus in the two Vanilla accessions CDY (cultivated on compost) and CLB (cultivated on leaf litter). This genus displayed thermophilic and thermotolerant properties and the sweating step may contribute to activate the multiplication of *Bacillus* strains. Xu et al. (2020) have described that the relative abundance of *Bacillus* changed during the curing process and the increased abundance of *Bacillus* may play an important role in the formation of vanilla aroma. It has been reported that some *Bacillus spp*. (*Bacillus siamensis* XY18 and *Bacillus subtilis* XY20) isolated from vanilla beans could increase the vanillin content of beans under *Bacillus*-assisted curing [17]. Besides, *Bacillus siamen*sis strains isolated from mature pods demonstrated a phytobeneficial effect and especially the ability to inhibit the growth of pathogens [27]. In addition to their role in the prevention of pathogens, some *Halomonas* spp. including *H. elongata* showed the ability to biotransform vanillin into ferulic acid [46]. *Halomonas* were also detected as the most abundant genera in other food products. A study on soybean pastes after fermentation showed that *Halomonas* was negatively correlated with isobutyric acid and ethyl butyrate accumulation, two compounds related respectively to acidic and fruity flavor. This genus was significantly present in dry Merlot red wines during the fermentation step and may contribute to enhance the aroma profile [47]. The implication of *Pseudoalteromonas* in the formation of volatile compounds has been established during the fermentation of shrimp paste: *Pseudoalteromonas* positively correlated with the formation of ketones, alcohols, esters, and acids [48]. To the best of our knowledge, no finding revealed the relationship between the genus *Carboxydocella* and the formation of aroma. The role of these four genera in the formation of volatile compounds needs to be studied in more detail.

No correlation was observed between the accessions or the substrate nature and the microbial diversity after the curing process. The changing environment during the curing process may explain the difference observed in the microbial profile for each accession. Indeed, the green pods of each accession were blanched (scalding step) separately in different batches according to the substrate nature and they were transferred in different wool blankets and wooden boxes for sweating.

## Conclusions

This study described for the first time the dynamic changes in the bacteria community associated to three vanilla accessions grown on two different substrates. The analysis carried out described the microbiota profile of each substrate; the bacteria internal transmission from the growing medium to the roots, and from the roots to the green pods. Each substrate is characterized by a unique diversity with Proteobacteria and Actinobacteria as the dominant phyla. The bacterial community appeared to differ according to the accession suggesting that the genotype of the plant influences the selection of bacteria in the soil. The analysis of endophytic bacteria in the roots showed that *Halomonas, Pseudoalteromonas, Enterobacter* and *Bradyrhizobium* were the most abundant genera in agreement with their phytobeneficial effect on the plant. *Halomonas, Pseudoalteromonas, Bacillus, and Carboxydocella* were found in the green pods and the beans after scalding and sweating. More than 250 aromatic molecules were identified in the aroma profile of Vanilla [1]. Even though several strains from the genus *Bacillus* demonstrated an effect on the formation of aromatic compounds, the other genera highlighted in this study might also contribute directly or indirectly to the formation of aromatic compounds. Their potential implication in the formation of aroma must be further investigated. This study describes bacterial community structure through the vanilla plant and is a preliminary study that highlights the dynamic transmission of bacteria from roots to beans and genera potentially involved in vanilla complex aroma.

## Methods

### Sample collection and preparation

*Vanilla planifolia* Andrews accessions were planted in a shade house in Sainte Suzanne, La Vanilleraie, La Réunion, France. Three accessions of *V. planifolia* (codified CDY, CLB and CHM) were selected by the owner of La Vanilleraie (Bertrand Côme), based on their vegetative growth, flowering capacity, and glucovanillin accumulation in green mature pods [20]. The identification of the plant material was undertaken by Bertrand Côme and a voucher specimen was deposited at the herbarium of the university of La Reunion. The voucher number of each accession is: CHM (REU 008544), CLB (REU 008545) and CDY (REU 008556). Since 2010, these accessions were cultivated on two growing media (compost and litter) to study the impact of the substrate type on the aromatic quality of the beans. The compost is fed with sugar cane bagasse, and the litter is composed of dead leaves of litchi trees as organic matter. Both substrates are supplemented once per year with either sugarcane bagasse or dead leaves. The soil supplied with compost was selected with reference to shade house cultivation, and the leaf litter (composed of dead leaves) referred to forest-type land cultivation. For each accession, all samples (rhizospheric growing media, roots, green pods, and green pods after scalding) were collected in July 2019 on the same day to reduce any heterogeneity imparted by climatic conditions. Three replicates were sampled on three *V. planifolia* plants for a total of 12 samples per accession. In more detail, the growing media and the roots were collected after removing compost or litter from the upper 10 cm layer of the soil. The roots were separated from the soil by rinsing three times in 500 mL of sterile water.

To study the community of endophytic bacteria of the roots, green pods and scalded green pods, the surface of all organs was sterilized. To this end, the samples were washed using sterile water, immersed for 10 min in sterile water containing 2.6% sodium hypochlorite, and washed again in sterile water for 10 min. All samples were stored at -80 °C for molecular analyses.

### DNA extraction

Samples were homogenized in liquid nitrogen. DNA was extracted by using two commercial DNA extraction kits. For soil samples, total DNA was extracted from 0.2 g soil using the PowerSoil Pro kit (Qiagen reference 47,014) according to the manufacturer’s instructions. For plant samples (roots, green pods, and green pods after scalding), total DNA was extracted from a 1 g sample using the DNeasy kit Plant Maxi (Qiagen reference 68,163) following the manufacturer’s instructions.

### 16 S rRNA gene sequencing

The V3-V4 region of the bacterial 16S rRNA gene was amplified using the primers 341F (5’-CCTACGGGNGGCWGCAG-3’) and 805R (5’-GACTACHVGGGTATCTAATCC-3’). These primers target a section of the bacterial 16 S rRNA region and generate amplicons of about 300 bp of length that is appropriate for Illumina sequencing. gDNA quantification, amplifications were performed in duplicate, PCR product purification, amplicon library construction and Illumina MiSeq sequencing (2 × 300 bp paired-end reads) were performed by Biofidal (Biofidal, Vaulx-en-Velin, France – (Pathania et al. 2020; Ruparelia et al. 2022)). A total of 19,901,711 reads were obtained and demultiplexed.

### Bioinformatics and statistical analysis

Quality control of the raw data was processed using FASTP [49]. Sequences were then processed using the DADA2 pipeline [50], which allows the construction of amplicon sequence variants (ASVs). Chimeric sequences were identified and removed. Taxonomy was assigned for 16 S using the Ribosomal Database Project (RDP) 16 S rRNA database (v. 11.5). ASVs tables were merged to the phyloseq project and processed as previously described [51, 52]. Statistical analyses were conducted using Rstudio [with phyloseq (v 1.42.0) [53], microbiome (v. 1.20.0) [54], Vegan (v. 2.6-4) [55], ggplot2 (v. 3.4.2) [56], DESeq2 (v. 1.38.3) [57], UpSetR (v. 1.4.0) [58] packages in R (version 4.2.3 (2023-03-15 ucrt)). Unique and shared bacterial ASVs were analyzed using UpsetR. Venn diagrams were constructed using Jvenn, a web-based tool (http://jvenn.toulouse.inra.fr/) [59].

Species richness and diversity indices were quantified using observed species, Shannon and Pielou evenness indices. Statistical comparisons were performed using the Wilcoxon rank-sum test, with asterisks indicating statistical significance. ns = non significatif; * *p*-value ≤ 0.05; ** *p*-value ≤ 0.01; *** *p*-value ≤ 0.001. Bray–Curtis and Jaccard β-diversity dissimilarities were used to characterize community structure and composition, respectively [60]. PCoA ordination was employed to depict community structure and composition differences between samples. Pairwise perMANOVA (Permutational multivariate analysis of variance) was conducted to assess the source of variation of β-diversity measures [61]. The bacterial datasets were also used to generate differential abundance plots between categories using the DESeq2 [62] with an alpha of 0.01 and fold change of 2 (log2 scale).

### Electronic supplementary material

Below is the link to the electronic supplementary material.

Supplementary Material 1

## Data Availability

16SRNA gene sequences are available on SRA database (BioProject ID: PRJNA1023005). All the other relevant data are provided in form of regular figures, tables, and Supplementary Material Files in the manuscript.
